# A Stochastic Approach to Generalized Modularity Based Community Detection

**DOI:** 10.3390/e27060554

**Published:** 2025-05-25

**Authors:** James Tipton, Jordan Langston

**Affiliations:** Department of Mathematics, Norfolk State University, Norfolk, VA 23504, USA; j.m.langston@spartans.nsu.edu

**Keywords:** community detection, generalized modularity, network analysis, 05C50

## Abstract

We study a stochastic approach to generalized modularity-based community detection by comparing two variants of the aforementioned approach to the standard modularity-based approach. In particular, we compare means and distributions. We also confirm that the stochastic approach can outperform standard modularity approaches.

## 1. Introduction

The community detection problem consists of identifying the community structure within a given network. This typically consists of providing a partition of the nodes in the given network. Here, we present a brief overview of the historical and recent developments of community detection.

### 1.1. Foundational Concepts and Early Methods

The notion of *community* in a graph was formalized in the early 2000s. In 2002, Girvan and Newman introduced the edge betweenness algorithm, which removes edges with the highest betweenness scores iteratively to uncover community structures [1]. The concept of modularity and its application to community detection was also introduced by Newman and Girvan in 2004 [2], and for larger networks by Clauset, Newman, and Moore in [3]. Modularity is a real number associated with a given partition that measures how strongly the partition divides the network into communities. A larger modularity score indicates higher edge density within individual communities and lower edge density between different communities. Communities are then detected by finding a partition that maximizes modularity. Modularity optimization became the basis for many subsequent algorithms, including the Louvain method [4], which remains widely used due to its scalability and performance on large networks. More recently, Quiring and Vassilevski presented a modularity-based community detection algorithm that can be run in parallel [5].

### 1.2. Spectral Clustering and Graph Partitioning

Spectral methods apply linear algebraic techniques to graph Laplacians to find clusters. These techniques are particularly powerful because they can detect community structures even when they are not apparent from the topology of the graph.

In [6], Shi and Malik used graph Laplacians and their eigenvectors for clustering in the context of image segmentation. In 2001, Ng, Jordan, and Weiss proposed a normalized spectral clustering algorithm that became foundational in machine learning [7]. In Section 2 we will review a spectral method based on [8] for optimizing generalized modularity.

### 1.3. Stochastic Methods in Community Detection

Stochastic approaches have become foundational in modeling and analyzing community structures within complex networks. These methods leverage probabilistic frameworks to capture the inherent randomness and uncertainty in real-world networks.

Stochastic block models are generative models for networks with a community structure. They provide a probabilistic framework in which each node belongs to a latent group, and the probability of an edge existing depends on the groups of the two nodes. Recent research includes Karrer and Newman’s work on degree-corrected stochastic block models, which handle degree heterogeneity in real-world graphs [9], and various works on exact recovery thresholds (see Abbe’s survey [10]), in which communities can be recovered perfectly as the size of the graph grows, especially under sparse regimes.

Further advancements include Bayesian formulations of stochastic block models, for example, Peng and Carvalho proposed a Bayesian degree-corrected stochastic block model using a logistic regression framework with node-specific correction terms [11].

### 1.4. Deep Learning and Graph Neural Networks

Recent trends have seen the incorporation of neural models for community detection. Graph autoencoders and variational autoencoders support learning embeddings that reveal latent community structure [12]. Graph convolutional networks have been used to classify nodes and indirectly infer communities [13]. Deep clustering combines embedding and clustering into a unified training objective [14,15]. Although powerful, these methods often require labeled data or heuristics and are less interpretable than classical methods.

### 1.5. Evaluation and Benchmarking

Evaluating community detection algorithms is nontrivial. Benchmarks like the Lancichinetti–Fortunato–Radicchi model [16] generate synthetic graphs with planted community structures and tunable parameters like overlapping nodes and community sizes. Real-world networks such as Zachary’s Karate Club are used extensively. Commonly used metrics include modularity, normalized mutual information, and the adjusted Rand index. Our focus here is on the modularity metric.

### 1.6. Applications in Real-World Networks

Community detection may be applied to a wide variety of networks, including social, biological, and information networks. For example, in scientific research networks, such as coauthorship or citation networks, community detection can uncover disciplinary boundaries, collaboration patterns, and the evolution of research fields; see the excellent survey [17] by Fortunato for more details. Although traditional community detection methods often rely on modularity maximization or spectral partitioning, motif-based methods such as those used in MEGA [18] highlight the potential of triangle-based clustering to capture tightly coordinated behaviors in social networks.

### 1.7. Generalized Modularity and Multiscale Community Detection

Modularity optimization remains a prevalent method for community detection, aiming to partition networks such that the density of edges within communities exceeds that expected in a null model. However, traditional modularity has limitations, notably the resolution limit, which hampers the detection of smaller communities. In fact, shortly after modularity was introduced, other methods of community detection arose using a modified version of Newman–Girvan modularity, see [19,20,21]. Such works led Fasino and Tudisco introduced a notion of generalized modularity in 2016 [22], which contained the aforementioned notions of modularity as special cases.

Generalized modularity frameworks have been developed to address the resolution limit. Lambiotte et al. [23] introduced a Markov stability approach which employs continuous-time Markov processes to evaluate the persistence of communities over multiple scales. This method supports community detection at varying resolutions. These generalized approaches often integrate stochastic dynamics, offering a dynamic perspective on community structure that complements static modularity measures.

### 1.8. Outline of the Paper

We continue the analysis of a community detection algorithm, based on modularity, developed in [24] which sought to explore a generalized modularity algorithm based on the algorithm given in [5]. It was noted in [24] that the use of generalized modularity allowed one to introduce an element of randomness to the algorithm. This stochastic version was shown to be capable of outperforming standard modularity algorithms. Here, we take a closer look at stochastic variants of the algorithm given in [24].

In Section 2, we provide a brief summary of the basic concepts needed to understand modularity-based community detection. Section 3 outlines the algorithm developed in [24] and our methodology to compare stochastic modularity algorithms to standard modularity. Our results indicate that generalized modularity algorithms can be tailored to the dataset at hand. These results are presented in Section 4. We conclude with a summary and possible avenues for future research in Section 5.

## 2. Community Detection Through Generalized Modularity

### 2.1. Generalized Modularity Matrices

Although there are many ways to perform community detection on a given network, it was Newman and Girvan [2] who introduced the notion of modularity as a means of community detection. Community detection in this way is performed by maximizing the modularity *Q* of the given network where

$$Q=\frac{1}{2m}\sum_{u=1}^{n}\sum_{v=1}^{n}\left(A_{uv}-\frac{k_{u}k_{v}}{2m}\right)\delta \left(c_{u},c_{v}\right),$$

with *m* the number of edges, *A* the adjacency matrix, and $c_{u}$ the community of vertex *u*. The matrix $B=A-\vec{k}\;{\vec{k}}^{T}/\left(2m\right)$ is called the modularity matrix of the network. In principle, community detection can be performed by finding the largest eigenvalue of the modularity matrix.

In 2018, Fasino and Tudisco generalized the notion of modularity [22]. This notion of generalized modularity includes as special cases the approaches given by Reichardt and Bornholdt [20], Ronhovde and Nussinov [21], and Arenas, Fernández, and Gómez [19].

**Definition** **1** ([22])**.** *A generalized modularity matrix is a matrix $M=A+W-\sigma \vec{k}\;{\vec{k}}^{T}\;$, where A is the adjacency matrix of an undirected, connected (and possibly weighted) graph, W a diagonal matrix, $\vec{k}$ a nonzero vector with nonnegative entries, and σ a positive real number.*

**Definition** **2.** *If M is a generalized modularity matrix and P is a partition of the vertices of the associated graph G=(V,E), then the associated modularity measure is*

$$Q=\sum_{i\in P}\left(e_{ii}-a_{i}^{2}\right),$$

*where $e_{ij}=\sum_{u,v\in V}\left(A_{uv}+W_{uv}\right)\delta \left(c_{u},i\right)\delta \left(c_{v},j\right)$, $a_{i}=\sqrt{\sigma }\sum_{u\in V}k_{u}\delta \left(c_{u},i\right)$, δ is the Kronecker delta, and $c_{u}$ and $c_{v}$ are the communities of u and v, respectively.*

It will be useful to note an equivalent formula for *Q*:

$$\begin{array}{cc} \sum_{i}\left(e_{ii}-a_{i}^{2}\right)\; & =\sum_{i}\left(\sum_{v,w}\left(A_{vw}+W_{vw}\right)\delta \left(c_{v},i\right)\delta \left(c_{w},i\right)-{\left(\sqrt{\sigma }\sum_{v}k_{v}\delta \left(c_{v},i\right)\right)}^{\;2}\right)\\ \; & =\sum_{i}\left(\sum_{v,w}\left(A_{vw}+W_{vw}\right)-\sigma \sum_{v,w}k_{v}k_{w}\right)\delta \left(c_{v},i\right)\delta \left(c_{w},i\right)\\ \; & =\sum_{v,w}\left(A_{vw}+W_{vw}-\sigma k_{v}k_{w}\right)\delta \left(c_{v},c_{w}\right). \end{array}$$

### 2.2. Spectral Interpretation

We conclude this section by showing that maximizing generalized modularity (in the case of two communities) corresponds to the largest eigenvalue of the generalized modularity matrix. We follow the argument presented in [24], which in turn is based on that in [8] for plain modularity matrices.

Let $M=A+W-\sigma \vec{k}\;{\vec{k}}^{T}$ be a generalized modularity matrix, and *i* a subset of the vertex set. The corresponding modularity measure is therefore

$$Q=\sum_{u=1}^{n}\sum_{v=1}^{n}\left(A_{uv}+W_{uv}-\sigma k_{u}k_{v}\right)\delta \left(c_{u},c_{v}\right).$$

Let $c_{a}$ and $c_{b}$ denote the two communities of the network, and define

sv=−1,v∈ca,−1,v∈cb.

Observe that

$$\begin{array}{cc} Q & =\sum_{u=1}^{n}\sum_{v=1}^{n}\left(A_{uv}+W_{uv}-\sigma k_{u}k_{v}\right)\delta \left(c_{u},c_{v}\right)\\ \; & =\frac{1}{2}\sum_{u=1}^{n}\sum_{v=1}^{n}\left(A_{uv}+W_{uv}-\sigma k_{u}k_{v}\right)\left(s_{u}s_{j}+1\right)\\ \; & =\frac{1}{2}{\vec{s}}^{T}\left(A+W-\sigma \vec{k}{\vec{k}}^{T}\right)\vec{s}+\frac{1}{2}\sum_{u=1}^{n}\sum_{v=1}^{n}\left(A_{uv}+W_{uv}-\sigma k_{u}k_{v}\right) \end{array}$$

since

$$\delta \left(c_{u},c_{v}\right)=\frac{1}{2}\left(s_{u}s_{j}+1\right)\;\mathrm{and}\;\frac{1}{2}\sum_{u=1}^{n}\sum_{v=1}^{n}\left(A_{uv}+W_{uv}-\sigma k_{u}k_{v}\right)\left(s_{u}s_{j}\right)=\frac{1}{2}{\vec{s}}^{T}\left(A+W-\sigma \vec{k}\;{\vec{k}}^{T}\right)\vec{s}.$$

We want the vector $\vec{s}$ that maximizes *Q* subject to the constraint $s_{i}=\pm 1$. Any $\vec{s}$ satisfying this constraint also satisfies ${\vec{s}}^{T}\vec{s}=n$. So, we instead consider the relaxed constraint ${\vec{s}}^{T}\vec{s}=n$ where $\vec{s}$ may point in any direction.

Proceeding with Lagrange multipliers, we have

$$\begin{array}{cccc} \; & \; & \frac{\partial }{\partial s_{i}}\left({\vec{s}}^{T}\left(A+W-\sigma \vec{k}\;{\vec{k}}^{T}\right)\vec{s}+\sum_{u=1}^{n}\sum_{v=1}^{n}\left(A_{uv}+W_{uv}-\sigma k_{u}k_{v}\right)-\lambda \left({\vec{s}}^{T}\vec{s}-n\right)\;\right) & =0\\ \; & \Leftrightarrow \; & 2\sum_{i=1}^{n}M_{ij}s_{j}-2\lambda \sum_{j}s_{j} & =0\\ \; & \Leftrightarrow & M\vec{s} & =\lambda \vec{s}. \end{array}$$

Thus, maximization of *Q* (in the relaxed setting) corresponds to the largest eigenvalue λ of M and is given by

$$Q=\frac{1}{2}\lambda {\vec{s}}^{T}\vec{s}=\frac{n\lambda }{2}.$$

The approximate solution (with the constraint $s_{i}=\pm 1$) is

si=−1,v→λ,i>0,−1,v→λ,i<0,

where ${\vec{v}}_{\lambda }$ is an eigenvector associated to λ.

## 3. Algorithm and Methodology

### 3.1. Generalized Modularity Framework

We use the algorithm devised in [24], which is essentially the algorithm presented in [5] only modified for generalized modularity matrices. For the reader’s convenience, we include an outline of the pertinent results from [24] here.

Recall that we have a generalized modularity $M=A+W-\sigma \vec{k}\;{\vec{k}}^{T}$ associated to some network *G* with vertex set *V* and edge set *E*, where *A* is an adjacency matrix, *W* a diagonal matrix, $\vec{k}$ a nonzero vector with nonnegative entries, and σ a positive number. Each partition of nodes for a given network corresponds to a declaration of communities within that network. Thus, each element of a given partition determines a community within the network. Given elements *i* and *j* of some partition *P* of vertices, denote the set union of *i* and *j* by (ij). The set (ij) can be thought to represent the community obtained by merging communities *i* and *j*. Denote the change in modularity *Q* from merging elements *i* and *j* by $\Delta Q_{\left(ij\right)}$. Finally, recall that for communities *i* and *j*, we define

$$e_{ij}=\sum_{u,v\in V}\left(A_{uv}+W_{uv}\right)\delta \left(c_{u},i\right)\delta \left(c_{v},j\right)\;\;\mathrm{and}\;\;a_{i}=\sqrt{\sigma }\sum_{u\in V}k_{u}\delta \left(c_{u},i\right).$$

**Lemma** **1** ([24])**.** *For all i,j,k∈P,*

$$e_{\left(ij)(ij\right)}=e_{ii}+e_{jj}+2e_{ij},\;e_{i(jk)}=e_{ij}+e_{ik}\;\mathrm{and}\;a_{\left(ij\right)}=a_{i}+a_{j}.$$

**Proof.**  Since (ij) consists of all vertices in *i* or *j*,

$$\begin{array}{cc} e_{\left(ij)(ij\right)}\; & =e_{ii}+e_{jj}+e_{ij}+e_{ji}=e_{ii}+e_{jj}+2e_{ij},\\ e_{i(jk)}\; & =\sum_{u,v}\left(A_{uv}+W_{uv}\right)\delta \left(c_{u},i\right)\delta \left(c_{v},\left(jk\right)\right)=\sum_{u,v}\left(A_{uv}+W_{uv}\right)\delta \left(c_{u},i\right)\left(\delta \left(c_{v},j\right)+\delta \left(c_{v},k\right)\right)=e_{ij}+e_{ik},\\ a_{\left(ij\right)}\; & =\sqrt{\sigma }\sum_{u\in (ij)}k_{u}\delta \left(c_{u},\left(ij\right)\right)=\sqrt{\sigma }\sum_{u\in i}k_{u}\delta \left(c_{u},i\right)+\sqrt{\sigma }\sum_{u\in j}k_{u}\delta \left(c_{u},j\right)=a_{i}+a_{j}. \end{array}$$

□

**Lemma** **2** ([24])**.** *For all i,j∈P, $\Delta Q_{\left(ij\right)}=2\left(e_{ij}-a_{i}a_{j}\right)$.*

**Proof.**  Let *P* be the original partition and $\tilde{P}$ be the partition after merging communities *i* and *j*. Applying Lemma 1, we find that

$$\begin{array}{cc} \Delta Q_{\left(ij\right)}\; & =\sum_{t\in \tilde{P}}\left(e_{tt}-a_{t}^{2}\right)-\sum_{t\in P}\left(e_{tt}-a_{t}^{2}\right)=e_{\left(ij)(ij\right)}-a_{\left(ij\right)}^{2}-\left(e_{ii}-a_{i}^{2}+e_{jj}-a_{j}^{2}\right)\\ \; & =e_{ii}+e_{jj}+2e_{ij}-{\left(a_{i}+a_{j}\right)}^{2}-\left(e_{ii}-a_{i}^{2}+e_{jj}-a_{j}^{2}\right)=2\left(e_{ij}-a_{i}a_{j}\right). \end{array}$$

□

**Lemma** **3** ([24])**.** *Let $\Delta Q_{P}$ be the matrix whose i,j entry is $\Delta Q_{\left(ij\right)}$ where i,j∈P. If i and (jk) are merged, then $\Delta Q_{\left(i(jk)\right)}=\Delta Q_{\left(ij\right)}+\Delta Q_{\left(ik\right)}$*

**Proof.**  By Lemmas 1 and 2,

$$\Delta Q_{\left(i(jk)\right)}=2\left(e_{i(jk)}-a_{i}a_{\left(jk\right)}\right)=2\left(e_{ij}+e_{ik}-a_{i}a_{j}-a_{i}a_{k}\right)=\Delta Q_{\left(ij\right)}+\Delta Q_{\left(ik\right)}.$$

□

### 3.2. Hierarchical Agglomeration Algorithm

**Theorem** **1** ([24])**.** *Let $P_{f}$ be a partition and $P_{c}$ a partition that is formed by merging elements of $P_{f}$. For $i\in P_{f}$, let $1_{i}$ be the vector whose vth entry is 1 if v∈i and 0 otherwise. If P is the matrix whose ith column is $1_{i}$, then*

$$\Delta Q_{P_{c}}=P^{T}\Delta Q_{P_{f}}P.$$

**Proof.**  Given $i,j\in P_{f}$ and $r,s\in P_{c}$, apply Lemmas 2 and 3 to find

$$Q_{\left(rs\right)}=\sum_{i\in r,j\in s}Q_{\left(ij\right)}=\sum_{i,j}Q_{\left(ij\right)}\delta \left(c_{i},r\right)\delta \left(c_{j},s\right),$$

showing that $Q_{\left(rs\right)}$ is the (r,s)-entry of $P^{T}\Delta Q_{P_{f}}P$. □

The above theorem underlies the following algorithm:

- Start with the adjacency matrix *A* of the network and the degree vector $\vec{k}$.
- Calculate the pointer vector $\vec{p}$ whose *i*th entry indicates which adjacent node the *i*th vertex would most like to merge with. This is the node for which $\Delta Q_{\left(ij\right)}$ is the largest positive value (ties can be broken by choosing the lowest such index *j*). If $\Delta Q_{\left(ij\right)}$ is nonpositive for all nodes *j*, then set $p_{i}=-1$. A very nice feature of this step is that it may be computed in parallel.
- Use the pointer $\vec{p}$ to merge nodes *i* and *j* that point to each other. Unpaired nodes remain as singletons.
- Construct the coarsening matrix *R* that has a row for each (possibly merged) node and the same number of columns as *A*. The *j*th entry in row *i* is 1 if node *j* was merged with node *i* and 0 otherwise.
- Calculate the coarsened adjacency matrix $A_{c}=RAR^{T}$ and the coarsened degree vector ${\vec{k}}_{c}=R\vec{k}$.
- The algorithm terminates if ΔQ contains no positive values. Otherwise, the algorithm recurs with $A_{c}$ and ${\vec{k}}_{c}$ in place of *A* and $\vec{k}$.

The above algorithm is slightly modified to use a randomized value of σ at each step. This modified algorithm is then used in two experiments aimed at comparing the performance of the stochastic generalized modularity algorithm to the performance of a standard modularity algorithm. Performance can then be compared by calculating the standard modularity measure for each partition. A higher measure indicates a better performance.

### 3.3. Experimental Setup and Evaluation Criteria

The first experiment runs a Reichardt–Bornholdt (RB) version of the stochastic algorithm 100 times and compares the mean modularity score to the modularity score obtained using the standard Newman–Girvan modularity algorithm. This is carried out over a variety of datasets. The Reichardt–Bornholdt modularity matrix M of a network is given by

$$M=A-\frac{\sigma }{2m}\vec{k}\;{\vec{k}}^{T},$$

where *A* is the adjacency matrix, 2m the number of edges, $\vec{k}$ the degree vector, and σ a positive number.

The second experiment runs the same Reichardt–Bornholdt algorithm and an Arenas–Fernández–Gómez (AFG) version of the stochastic algorithm 100 times each and compares means. This is also carried out over a variety of datasets. The Arenas–Fernández–Gómez modularity matrix M is given by

$$M=A+\sigma I-\frac{\left(k+\sigma \vec{1}\right){\left(k+\sigma \vec{1}\right)}^{T}}{\sigma n+2m},$$

where *n* is the number of vertices of the graph and σ is a real number.

We pause to address an important point about how the resulting partitions are compared. Given some network *G*, running an RB modularity algorithm will return some partition, say $P_{1}$, which is found by optimizing the associated RB modularity measure. Similarly, running an AFG modularity algorithm will return partition $P_{2}$, which is found by optimizing the associated AFG modularity measure. Finally, we compare the partitions $P_{1}$ and $P_{2}$ by calculating their corresponding Newman–Girvan modularity scores.

In our experiments, we focus on two representative generalized modularity frameworks: the Reichardt–Bornholdt (RB) and Arenas–Fernández–Gómez (AFG) models. Although arguably contrived, these models were selected because of their prominence in the literature and their tunable resolution parameters, which allow for a direct investigation of how randomized parameter selection affects performance within the generalized modularity framework.

### 3.4. Stability Under the Stochastic Parameter σ

To assess the sensitivity of the stochastic generalized modularity approach to the parameter σ, we conducted a stability analysis on the datasets ENZYMES-g479 (small biological network), G11 (medium-sized synthetic network), and fe-4elt2 (large sparse graph), using RB modularity. For each dataset, we performed 100 runs with σ uniformly distributed over the intervals (0, 0.5), (0.5, 1.5), and (1.5, 2.5).

We recorded the (standard) modularity score, number of communities, normalized variation of information (NVI) between partitions, and runtime. NVI returns 0 if the two partitions are identical and 1 if completely different, see [25] for more details. These metrics allow us to evaluate both the performance and consistency of the algorithm under varying parameter spaces. The results of this analysis are presented in Section 4.

## 4. Results

### 4.1. Performance on Benchmark Datasets

Looking at Table 1, generalized modularity outperforms or ties standard modularity on every dataset tested except econwm2. More importantly, generalized modularity has the ability to outperform standard modularity on average for certain datasets, take for example, ca-netscience. The economics dataset econwm2 is an interesting case: RB modularity did not outperform standard modularity a single time, unlike AFG modularity. This supports the idea of tailoring a generalized modularity algorithm to the dataset at hand.

**Figure 1:**
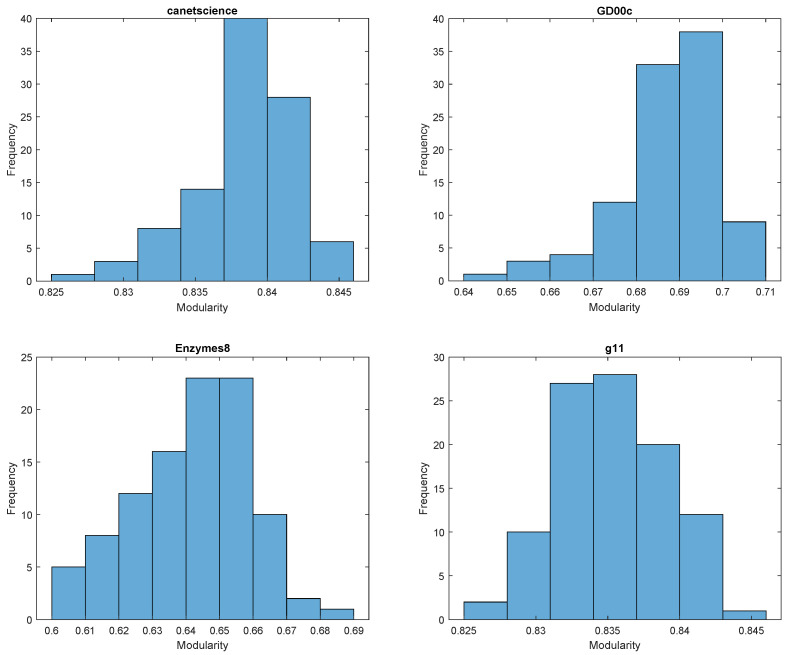
Histograms of stochastic RB modularity with 100 cycles with σ uniformly distributed in (0.5, 1.5). The distributions appear to follow a slightly skewed normal distribution.

**Figure 2:**
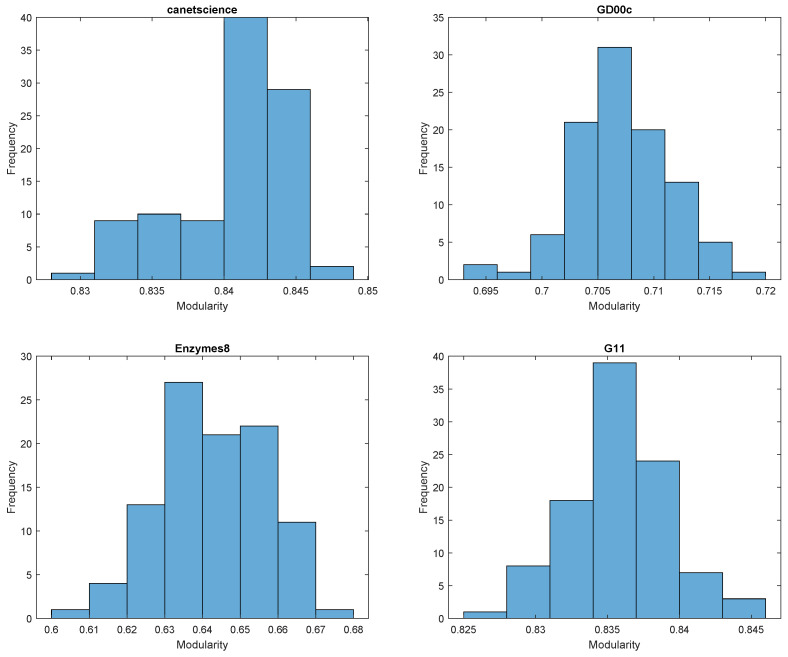
Histograms of stochastic AFG modularity with 100 cycles with σ uniformly distributed in (0.5, 1.5). The distributions appear to follow a roughly normal distribution.

In Figure 1, the histograms for stochastic RB modularity are all skewed, with canetscience, GD00c, and G11 negatively skewed and Enzymes8 positively skewed. In Figure 2, the histograms for the same datasets but with stochastic AFG modularity appear to follow the normal distribution more closely, each showing some improvement in symmetry. Although it is clear that canetscience is still negatively skewed.

Notably, the standard deviations reported in Table 1 are small for both methods, indicating stability, but RB tends to have slightly higher variability, consistent with the stronger skew observed in its distributions. AFG modularity results are not only more symmetric but also show tighter ranges, suggesting more consistent outputs.

Execution times reported in Table 1 reveal that both methods scale with dataset size. However, AFG generally completes slightly faster than RB on large datasets, as seen in GD00-c and fe-4elt2, which could be a practical advantage in large-scale applications.

### 4.2. Distributional Insights and Output Variability

A two-sample *t*-test was performed on the stochastic RB and AFG modularity scores, with a null hypothesis of equal means. This null hypothesis is rejected for ca-netscience and GD00-c but not for Enzymes8 nor G11. This result again supports the idea that one generalized modularity matrix may be better suited to a given dataset than others. For example, RB modularity outperforms AFG modularity on ENZYMES-g479 but not on ENZYMES8. Interestingly, this occurs despite the more symmetric and consistent distributions of AFG, showing that symmetry does not always translate to superior performance.

In addition to outperforming or matching standard modularity in most cases, the stochastic framework allows us to observe variability in modularity outcomes across runs. The distributional statistics and histograms reported in Table 1 and Figure 1 and Figure 2, respectively, illustrate that different generalized modularity formulations (e.g., RB vs. AFG) produce distinct variability profiles across datasets. This suggests that the stochastic method does more than help escape local optima—it provides a mechanism for comparing the consistency of results under different models. Although our current analysis focuses on modularity values, future work may investigate the structure of the resulting ensemble of partitions, offering further insight into the stability and robustness of community assignments.

The performance differences observed across datasets can likely be attributed to interactions between the network structure and the modularity formulation. For example, the poor performance of RB on econwm2 may be due to its high edge density and complex topology, which could misalign with the resolution scale favored by RB’s default parameter range. Conversely, datasets such as ca-netscience and Enzymes-g479 show improved performance under stochastic RB, suggesting that RB’s formulation is well-suited to sparser, modular graphs when variance is introduced through σ. The AFG formulation tends to yield more stable outputs, possibly due to its inherent weighting scheme, which smooths variability across iterations. A more comprehensive structural characterization of each dataset, such as community strength, assortativity, or degree heterogeneity, would help ground these interpretations.

### 4.3. Observations from Parameter Sensitivity Analysis

The stability analysis reveals a consistent trend: performance and stability of the stochastic algorithm vary significantly depending on the distribution of σ and network characteristics. For ease of exposition, we will denote the mean, variance, minimum, and maximum of the normalized variation of information by an ordered quadruplet.

For the dataset ENZYMES-g479:

- For the interval (0, 0.5), the summary statistics were (0.2043, 0.0157, 0, 1) and the total runtime was about 33 s. The algorithm suffers from moderate instability in this case. Some runs produce identical partitions while others produce completely different partitions. Furthermore, some runs put all nodes into a single community while others made every node its own community. The parameter range in this case is too low to meaningfully resolve stable communities in this network.
- For the interval (0.5, 1.5), the summary statistics were (0.2308, 0.0051, 0, 0.4313) and the total runtime was about 22 s. Although the NVI is slightly higher, the variance and range are much tighter. Thus, we have more consistent partitions despite the slightly higher average dissimilarity. In fact, 64 of the 100 runs produced partitions with 4 communities with low variance in modularity, further supporting that this case yields high-quality and stable partitions.
- For the interval (1, 2.5), the summary statistics were (0.1979, 0.0028, 0, 0.3734) and the total runtime was about 21 s. Here we have a lower mean and variance in NVI, hinting at more consistent partitioning than the first case. However, the average modularity found here was 0.1814, much lower than the modularity found in the previous case (0.5339). The average community number was 17. Thus, even though the partitions are more stable, they are consistently over-partitioned and of lower quality in terms of modularity.

For the dataset G11:

- For the interval (0, 0.5), the summary statistics were (0.0712, 0.0163, 0, 0.5386) and the total runtime was about 4.5 min. In this case, the low mean NVI suggests that partitions are fairly similar on average across runs. However, the high variance and wide max indicate that some runs produce very different partitions. In fact, every run produced a single community except for one run, which produced 36 communities. This suggests instability and poor resolution at low σ. Most runs will collapse the network into a single community, while an occasional run will over-partition it.
- For the interval (0.5, 1.5), the summary statistics were (0.1647, 0.0002, 0.0982, 0.2076) and the total runtime was about 2.7 min. Though the NVI is higher compared to the previous case, here it is tightly concentrated with low variance. In this case the stochastic algorithm consistently finds moderately distinct but structurally similar partitions. The modularity itself is very high and stable with a mean of 0.8305 and variance of about 0.00003. Furthermore, the number of communities is narrowly ranged (mean of 10.85, min of 8, max of 14). Here, the algorithm achieves both quality and consistency, making it the best-performing configuration for G11.
- For the interval (1.5, 2.5), the summary statistics were (0.2792, 0.0001, 0.2309, 0.3163) and the total runtime was about 1.5 min. The NVI is higher than both previous cases. Even though the variance is low, the entire distribution is shifted upward, indicating consistently more dissimilar partitions. The number of communities is also quite high, with a mean of 49, suggesting over-partitioning. The average modularity 0.7115 remains high but is noticeably lower than the previous case. In this case, the partitions are more fragmented and experience slightly degraded modularity.

For the dataset fe-4elt2:

- For the interval (0, 0.5), the summary statistics were (0.0213, 0.0043, 0, 0.3573) and the total runtime was about 38 h. The terrible runtime is bad enough, but worse yet, the results are not worth the wait. Again, the low mean NVI is misleading due to almost all runs yielding the single community partition. The modularity is extremely low as a result, confirming that the output is trivial and structurally uninformative for this parameter range in the case of large, sparse graphs like fe-4elt2.
- For the interval (0.5, 1.5), the summary statistics were (0.1877, 0.00008, 0.1549, 0.2150 and the total runtime was about 21 min. The NVI values are moderate but tightly clustered, suggesting the algorithm consistently finds distinct but structurally similar partitions. The high average modularity (0.8992), together with the low variance in modularity (0.000007) and a reasonable community count distribution (20.21 on average with a range of (16, 26) indicates high-quality and stable outputs. This interval produces both meaningful and robust results, making it the most effective for fe-4elt2.
- For the interval (1.5, 2.5), the summary statistics were (0.2045, 0.00002, 0.191, 0.2172) and the total runtime was about 10 min. The NVI is slightly higher than the previous case, but still very tightly concentrated. In this case, though, the algorithm over-partitions the network (112 communities on average), with modularity remaining fairly high (0.873,) though lower than the previous case. Results are consistent but overly granular. This could indicate overfitting structure, or perhaps the detection of substructures within the optimal communities.

**Figure 3:**
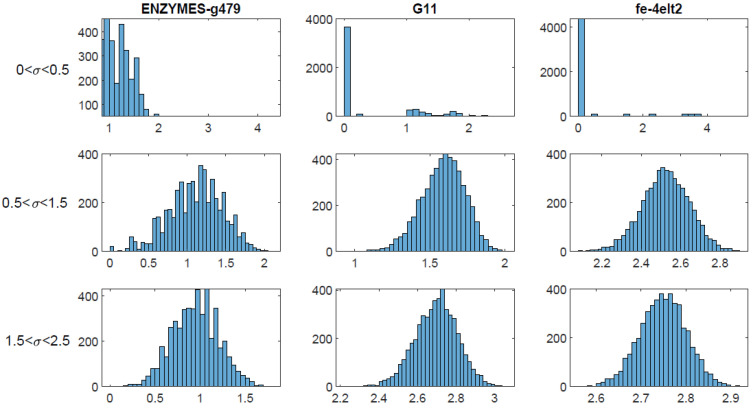
Histograms of normalized variation of information values for the networks ENZYMES-g479, G11, and fe-4elt2 with σ normally distributed in (0, 0.5), (0.5, 1.5), and (1.5, 2.5), using stochastic RB modularity. The first row shows poor performance across all datasets, while the second and third rows indicate varying degrees of stability.

Refer to Figure 3 to see histograms of the NVI in each of the above cases. These results indicate that parameter choice should be tuned to the network structure. Lower ranges for σ may lead to under-partitioning in large or dense graphs, while moderate ranges can yield stable, high-quality results. This supports the inclusion of adaptive or data-driven range selection for σ in future implementations.

## 5. Future Work and Conclusions

Our study demonstrates that stochastic generalized modularity algorithms can outperform traditional modularity-based methods on certain datasets. By introducing randomness into the resolution parameter σ at each step of a hierarchical coarsening process, we observe measurable gains in modularity scores and expose variation in outcome stability across model types. These findings suggest that generalized modularity methods can be flexibly adapted to the structure of a given network, rather than applying a single global approach.

Several avenues remain open for expanding the theoretical and empirical scope of this work:

First, a rigorous analysis of the asymptotic behavior of the stochastic generalized modularity algorithm is needed. Understanding how convergence rates, complexity, and partition quality scale with network size could offer important guarantees and practical design guidelines.

Second, it is important to analyze the behavior of the algorithm on more structurally diverse networks. This includes networks with highly overlapping communities, heterogeneous degree distributions, and hierarchical or multilayer structures. In particular, exploring the adaptability of the stochastic method to dynamic networks—where community structure evolves over time—and very large-scale graphs would extend the method’s practical relevance.

Third, while we demonstrate variability in modularity outcomes across stochastic runs, further investigation is needed into the ensemble of resulting partitions. Metrics such as pairwise partition similarity, co-association frequency, and consensus clustering could be used to assess robustness and extract stable community cores.

Finally, expanding the theoretical foundation of the method, including a more principled treatment of how parameter randomization influences the optimization landscape, would clarify under what conditions the stochastic method is likely to outperform deterministic alternatives.

In conclusion, this work represents an initial step towards a more flexible and data-sensitive approach to modularity-based community detection. We anticipate that further refinement and theoretical grounding will enhance both the performance and interpretability of the stochastic framework.

## Figures and Tables

**Table 1:** Standard modularity (*Q*) and summary statistics (mean, (min, max), standard deviation) and time (in seconds) for the stochastic RB and AFG methods.

Dataset	Nodes, Edges	*Q*	$Q_{\mathrm{RB}}$	$Q_{\mathrm{AFG}}$	$t_{\mathrm{RB}}$	$t_{\mathrm{AFG}}$
ENZYMES-g479	28, 49	0.5121	0.5342, (0.3898, 0.5854), 0.0265	0.5233, (0.4898, 0.5591), 0.0148	0.2590	0.2298
ENZYMES8	88, 133	0.6427	0.6419, (0.6022, 0.6809), 0.0169	0.6435, (0.6075, 0.6715), 0.0137	0.4104	0.3903
ca-netscience	379, 914	0.8359	0.8384, (0.8265, 0.8453), 0.0036	0.8408, (0.8307, 0.8461), 0.0038	0.7537	0.7140
GD00-c	638, 1025	0.7130	0.7061, (0.6891, 0.7158), 0.0055	0.7066, (0.6964, 0.7147), 0.0040	1.6244	1.4521
G11	800, 1600	0.8382	0.8352, (0.8274, 0.8440), 0.0036	0.8357, (0.8276, 0.8448), 0.0035	0.93175	0.8392
fe-4elt2	11K, 33K	0.9055	0.9050, (0.9005, 0.9088), 0.0015	0.9066, (0.9043, 0.9090), 0.0010	10.6930	10.0515
econwm2	260, 2.8K	0.2437	0.1805, (0.1081, 0.2106), 0.0198	0.2378, (0.2110, 0.2496), 0.0072	3.2079	3.4837
road-usroads	129K, 165K	0.9784	0.9778, (0.9773, 0.9784), 0.00022881	0.9778, (0.9772, 0.9783), 0.00021718	213.9985	213.1294

## Data Availability

The network data presented in this study are openly available in the Network Data Repository at doi/10.5555/2888116.2888372. All other raw data supporting the conclusions of this article will be made available by the authors on request.
